# Comprehensive analysis revealed P4Hs as new biomarkers for prognosis and immunotherapy in head and neck cancer

**DOI:** 10.1038/s41598-024-62678-9

**Published:** 2024-05-28

**Authors:** Huan Zhou, Yulin Lei, Jing Luo, Jianmei Wang, Lin Peng, Kelin Mou, Li Xiang, Yuhao Luo

**Affiliations:** 1 Department of Oncology, The Affiliated Hospital of Southwest Medical University, Luzhou, China; 2 Department of Cardiology, The Affiliated Hospital of Southwest Medical University, Luzhou, China; 3 Department of Pathology, The Affiliated Hospital of Southwest Medical University, Luzhou, China; 4 Department of Bone and Joint, The Affiliated Hospital of Southwest Medical University, Luzhou, China; 5 Department of Oncology, The Affiliated Tianfu Hospital of Southwest Medical University, Meishan, China

**Keywords:** Bioinformation analysis, HNSC, Immune infiltration, P4HA1-3, Prognostic biomarkers, Cancer, Immunology

## Abstract

Prolyl 4-hydroxylases (P4Hs) are a family of key modifying enzymes in collagen synthesis. P4Hs have been confirmed to be closely associated with tumor occurrence and development. However, the expression of P4Hs in head and neck cancer (HNSC) as well as its relationship with prognosis and tumor immunity infiltration has not yet been analyzed. We investigated the transcriptional expression, survival data, and immune infiltration of P4Hs in patients with HNSC from multiple databases. P4H1-3 expression was significantly higher in HNSC tumor tissues than in normal tissues. Moreover, P4HA1 and P4HA2 were associated with tumor stage, patient prognosis, and immune cell infiltration. P4HA3 was related to patient prognosis and immune cell infiltration. Correlation experiments confirmed that P4HA1 may serve as a prognosis biomarker and plays a role in the progression of nasopharyngeal carcinoma. These findings suggest that P4HA1-3 may be a novel biomarker for the prognosis and treatment of HNSC, which is expected to support the development of new therapies for patients with head and neck tumors and improve patient outcomes.

## Introduction

Prolyl hydroxylation is a universal posttranslational modification that plays an important role in regulating protein folding and stability in mammalian cells^1–3^. Prolyl 4-hydroxylases (P4Hs), a family of enzymes present in the endoplasmic reticulum, play a key role in collagen biosynthesis^4^. Numerous studies have shown that P4Hs are α2β2 tetramers consisting of two α subunits and two β subunits. The α subunits contain the catalytic site of the enzyme and the peptide substrate binding domain, and the β subunits are protein disulfide isomerases^3,5^. Three prolyl-4-hydroxylase α isomers have been identified in mammals: P4H alpha 1(P4HA1), P4H alpha 2 (P4HA2), and P4H alpha 3 (P4HA3)^1^. Previous studies have found that P4Hs are dysregulated in a variety of tumors, including melanoma, breast cancer, lymphoma, esophageal cancer, pancreatic cancer, lung cancer, and prostate cancer^6–12^. P4Hs are closely related to tumor proliferation, invasion, and metastasis, and play important roles in promoting cancer development^1,9,13,14^. Kaluz et al. found that targeting HIF-activated collagen proline 4-hydroxylase expression reduced collagen deposition, thereby inhibiting melanoma growth^15^. Glikes DM et al. reported that increased expression of P4HA1 mediated by hypoxia-inducing factor HIF-1α could promote the invasion and metastasis of breast cancer cells and is associated with poor prognosis in breast cancer^11^. Feng G et al. found that miR-30e could inhibit the proliferation of liver cancer cells by targeting P4HA1^16^. In vitro and in vivo studies have confirmed that P4HA2 can regulate the PI3K/Akt/mTOR signaling pathway to promote the occurrence and development of liver cancer^17^.Similarly to P4HA2, P4HA3 can mediate clear cell renal cell carcinoma progression by regulating the PI3K/AKT/GSK3β pathway^18^.

In vitro and in vivo studies have confirmed that P4HA2 can regulate the PI3K/Akt/mTOR signaling pathway to promote the occurrence and development of liver cancer^19,20^. Many studies have shown that the occurrence of HNSC is related to smoking, alcohol use, Epstein–Barr virus infection, and human papillomavirus infection, among other factors^21^. HNSC exhibits a highly heterogeneous tumor microenvironment, that can achieve immune escape through a variety of mechanisms^22^. While advances in treatment have improved prognosis in patients with HNSC, many cases still involve metastasis and exhibit resistance to various therapies^23–26^. Therefore, it is necessary to explore new biomarkers of HNSC prognosis and treatment.

In this study, we aimed to visualize the prognostic values of P4Hs in HNSC and investigate the relationship between P4Hs expression and immune infiltration by using different databases such as The Cancer Genome Atlas (TCGA) database, the Genotype–Tissue Expression Database (GTEx), Gene Expression Profiling Interactive Analysis (GEPIA), Kaplan–Meier Plotter, cBioPortal databases, and Tumor Immune Estimation Resource (TIMER). Bioinformatics analysis indicate that P4Hs are novel oncogenes associated with prognosis and immune infiltration. Moreover, we confirmed that P4HA1 was associated with poor prognosis in nasopharyngeal carcinoma and could promote proliferation and metastasis in nasopharyngeal carcinoma. These findings indicate that P4Hs may be clinical therapeutic targets for HNSC.

## Materials and methods

### Data collection

Gene expression data were obtained from TCGA (https://portal.gdc.cancer.gov/) and GTEx (https://gtexportal.org/) databases. All RNA-seq data were obtained in the format of fragments per kilobase of exon model per million mapped reads or transcripts per kilobase of exon model per million mapped reads normalized.

### Gene expression analysis

In the Xiantao Academy tool (https://www.xiantao.love/) module, we input P4H “differences in gene expression analysis” to observe differences in P4Hs expression between different tumors or specific tumor subtypes and adjacent normal tissues in TCGA and GTEx; log2(x + 0.001) transformation was performed for each expression value. Moreover, we eliminated cancer species with less than 3 samples for a single species and obtained the expression data of 34 total cancer species. We also obtained the expression difference between tumor tissues and corresponding normal tissues. Using the “Expression DIY-Profile” and “Expression DIY-Box Plots” modules of GEPIA2 (http://gepia2.cancer-pku.cn/#index/) with P-value cutoff = 0.01, log2FC (fold change) cutoff = 1, and “Match TCGA normal and GTEx data,” we obtained tumor tissues with GTEx data corresponding to normal tissue expression differences of box chart. In addition, we used SangerBox (http://www.sangerbox.com/tool), a clinical stage and pathological expression analysis module, to obtain different pathological TCGA tumor stages (phase I, II, III and IV) and develop violin plots. The Human Protein Atlas (https://www.proteinatlas.org) database was also applied to validate P4H levels in HNSC.

### Survival analysis

We used the “gene expression prognosis analysis” module in SangerBox to obtain the overall survival (OS) and progression-free interval (PFS) significance map data of P4Hs in HNSC. The website used the R package maxstat (Maximally selected rank statistics with several p-value approximations version: 0.7–25) to calculate the optimal cut-off values for ENSG00000122884 (P4HA1), ENSG00000072682 (P4HA2), and ENSG00000149380 (P4HA3). The settings were that the minimum number of samples in a group should be greater than 25% and the maximum number of samples in a group should be less than 75%. After obtaining the optimal cut-off values, the patients were divided into high and low groups based on these values. Furthermore, the R package survival's survfit function was used to analyze the prognostic differences between the two groups. The logrank test method was then applied to evaluate the significance of prognostic differences among different groups, resulting in the final observed prognostic differences. The logrank test was used to evaluate the significance of prognostic differences among different groups, and a tree of risk rates was drawn.

### Gene mutation analysis

Using cBioPortal (https://www.cbioportal.org/), we selected “TCGA Pan Cancer Atlas Studies” in the “Quick the select module” and entered “P4Hs” to query P4H genetic change characteristics. The change frequency, mutation type, and CNAs of P4Hs in HNSCS were observed in the “Cancer Types Summary” module, and the correlation between P4Hs was compared. Differential mutation patterns were identified using Fisher's exact test, and genes with p value less than 0.05 were defined as differentially mutated genes^27^. Due to copyright, we are unable to obtain the internal R code.

### Enrichment analysis of P4Hs-related genes

We entered “P4HA1,” “P4HA2,” and “P4HA3” into the search bar of GeneMANIA (http://www.genemania.org) to identify the 20 genes most associated with the P4Hs.

We used GEPIA2's “Similar Gene Detection” module to obtain the first 100 P4HA1, P4HA2, and P4HA3–related genes based on all TCGA tumor datasets. Kyoto Encyclopedia of Genes and Genomes (KEGG) analysis was performed in the “GO and KEGG one-bonding enrichment analysis tool” module in SangerBox.

### Immune infiltration analysis

The “immune infiltration analysis” module of the Xiantao Academy tool was entered, and the three P4Hs genes were input to obtain the “immune infiltration lollipop map.” We used the “Gene—Immune Infiltrates” module in TIMER (https://cistrome.shinyapps.io/timer/) to explore the relationship of P4H expression with immune infiltration in HNSC. B Cell CD8 + T Cell, CD4 + T Cell, Macrophage Neutrophil, and Dendritic Cell were selected to obtain immunoinfiltration scatter plots. In addition, we also obtained the expression map of P4H immune checkpoint gene in HNSCS through the immune checkpoint gene analysis module in SangerBox. We also filtered all normal samples and further performed a log2(x + 0.001) transform for each expression value. Next, we calculated Pearson correlations between theP4Hs and marker genes of five immune pathways.

### Cell lines and culture

Human nasopharyngeal carcinoma cell line 5-8F was purchased from Sichuan Spolikang Biotechnology Co., LTD. All 5-8F cells were cultured in RPMI-1640 medium (Hyclone, Logan, UT, USA) with 10% fetal bovine serum (FBS) (GIBCO BRL, Invitrogen, Grand Island, NY, USA). All cell lines were incubated under an atmosphere of 5% CO2 at 37 °C.

### Tumor tissue samples

All tumor tissues were provided by the Tumor Tissue Bank of The Affiliated Hospital of Southwest Medical University. All specimens were attached to a confirmed pathological diagnosis. All experiments were endorsed by the Ethics Committee of The Affiliated Hospital of Southwest Medical University and complied with the Declaration of Helsinki.

### Construction of sh-RNA, and cell transfection

These assays were performed according to previously described methods^28^. The shRNAs sequences were list at Supplementary Table S1.

### Real-time qPCR and western blot assays

Real-time qPCR and western blot assays were performed according to previously described methods^28^. In western blot assays, instead of using the whole membrane to transfer the protein, we transfer the protein after cutting the gel according to the molecular weight of the target protein. All primers designed for qPCR are listed in Supplementary Table S2. The details of antibodies used for western blot assay are listed in Supplementary Table S3.

### CCK‑8 assay

We seeded cells in 96-well plates at 1.8 × 103 per well in 100 μl of complete medium and 10 μl of CCK-8 reagent (Sichuan Spolikang Biotechnology Co., LTD) for 1.5 h each day after 5 days of culture. We then used a microplate to measure the absorbance of each well at 450 nm. Each sample was tested three times.

### Colony formation assay

We seeded cells in 6-well plates at 1500 cells per well and cultured them in complete medium. After 2 weeks, colonies were stained using crystal violet (Sichuan Spolikang Biotechnology Co., LTD) for 30 min and washed with phosphate buffered saline two times. We then counted colonies with diameters greater than 1 mm.

### Transwell assay

We seeded 3 × 104 cells in the upper layer of the transwell membrane, while the lower chamber contained 10% FBS to induce cell migration. The transwells were incubated at 37 °C under an atmosphere with 5% CO2 for 48 h, the upper layer of the transwell membrane was wiped, and the cells that passed through the membrane were stained with crystal violet for 30 min and observed by microscopy.

## Results

### Transcriptional levels of P4Hs expression in HNSC

**Figure 1:**
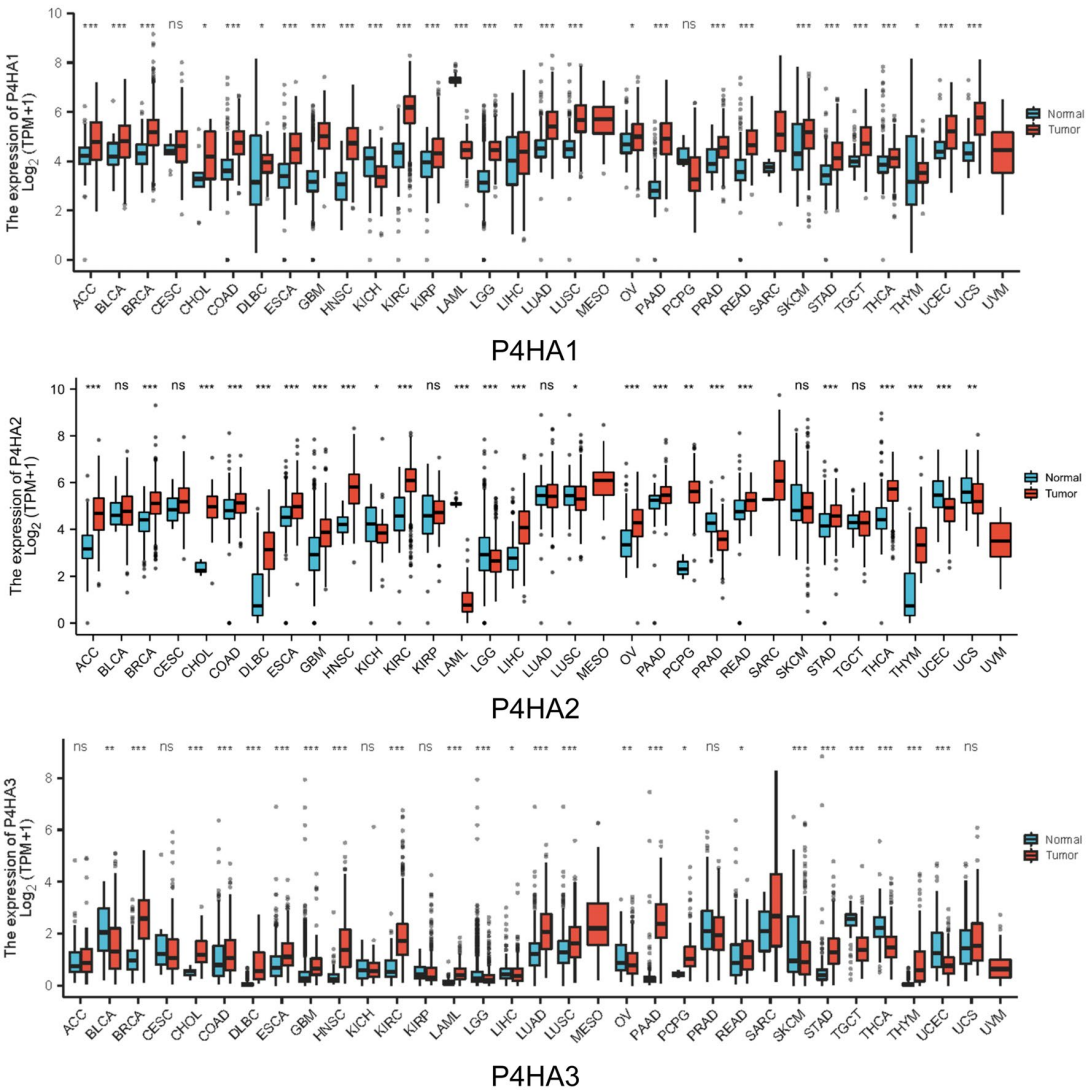
Expression level of P4Hs in tumor and normal tissues. The Xiantao Academy tool was used to analyze P4HA mRNA expression in pancarcinoma tissues and corresponding control tissues. Tumors and normal tissue are shown in red and blue, respectively.

We used TCGA databases to assess how P4H expression differed in tumors and correlated normal tissue samples. Compared with normal controls, the expression levels of P4HA1, P4HA2, and P4HA3 were significantly higher in tumor tissues, particularly in HNSC (Fig. 1).

### Relationship between P4Hs and the clinicopathological parameters in HNSC

**Figure 2:**
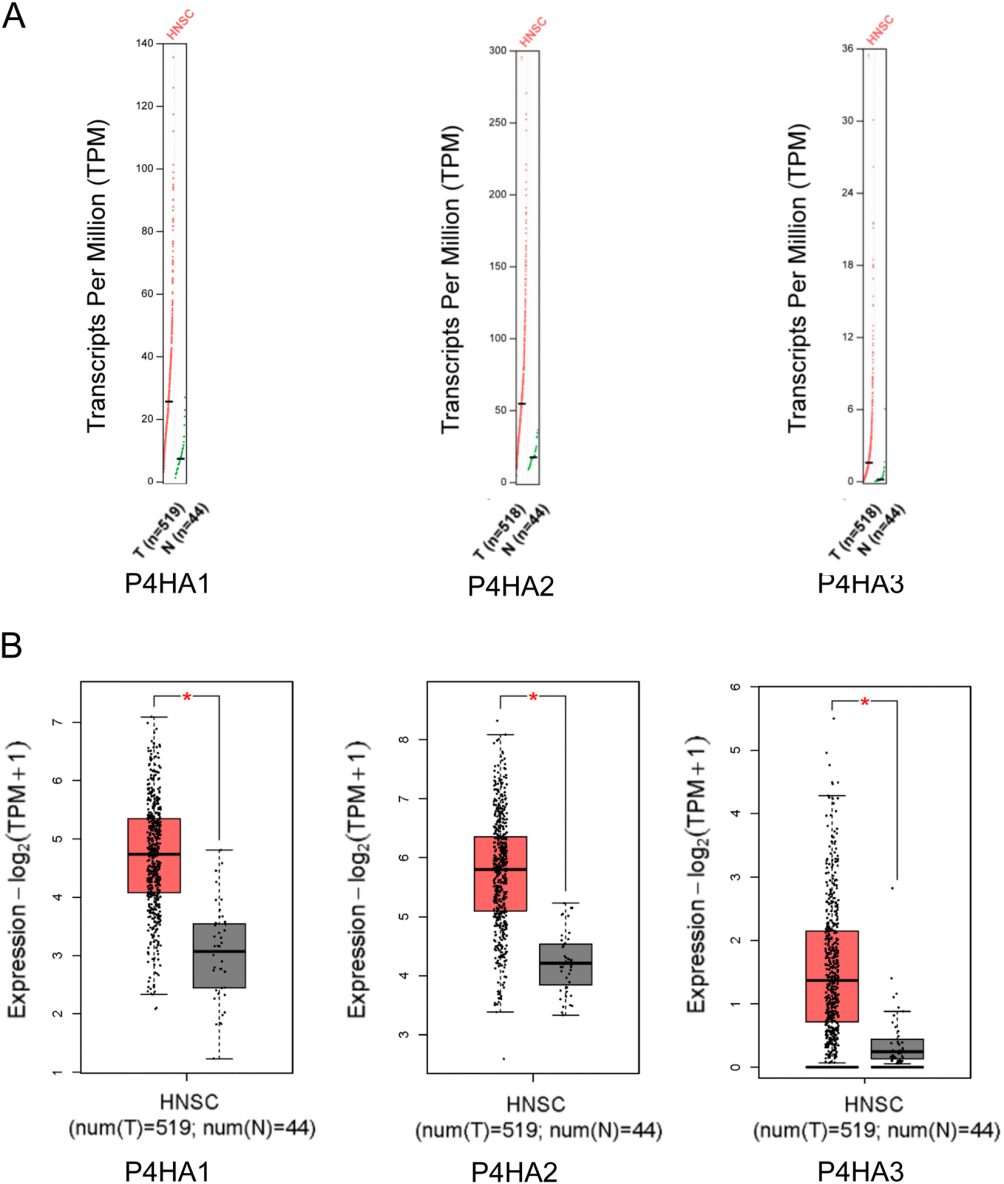
High expression of P4Hs in HNSC. (**A**), (**B**) P4H expression was higher in HNSC than in normal tissues, as determined using the GEPIA2 profile and BOX plot analysis module.

**Figure 3:**
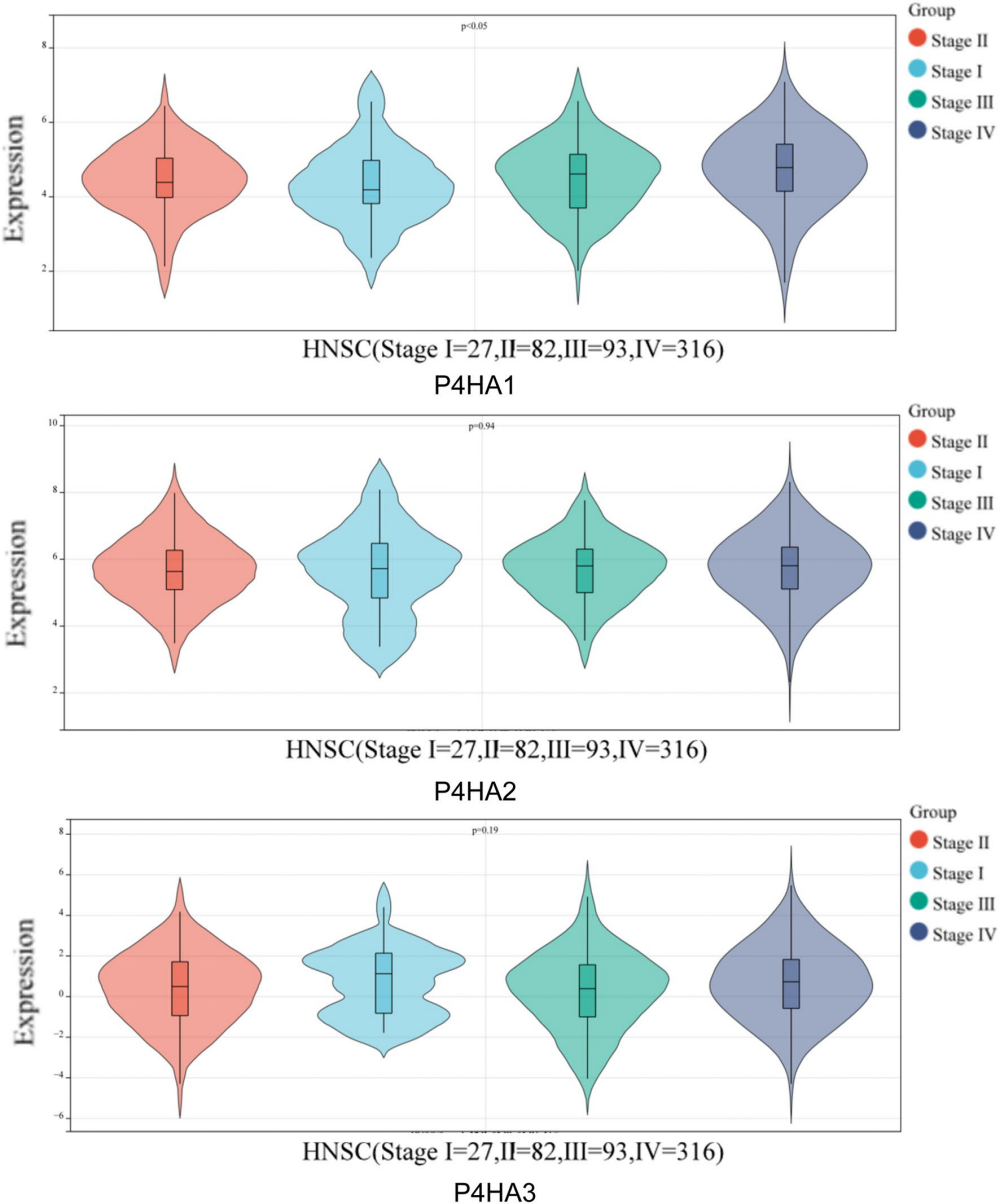
Expression levels of P4Hs in different stages of HNSC4 were analyzed using the SangerBox tool.

We compared the mRNA expression of P4Hs between HNSC and normal tissues. The expression levels of P4HA1, P4HA2, and P4HA3 were higher in HNSC than in normal tissues (Fig. 2A, B). In addition, we analyzed the relationship between P4H expression and HNSC tumor stage for HNSC. We found that, compared to normal tissues, P4HA1 expression was significantly elevated in tumor stages III, IV. Whereas P4HA2 and P4HA3 groups did not significantly differ (Fig. 3).

**Figure 4:**
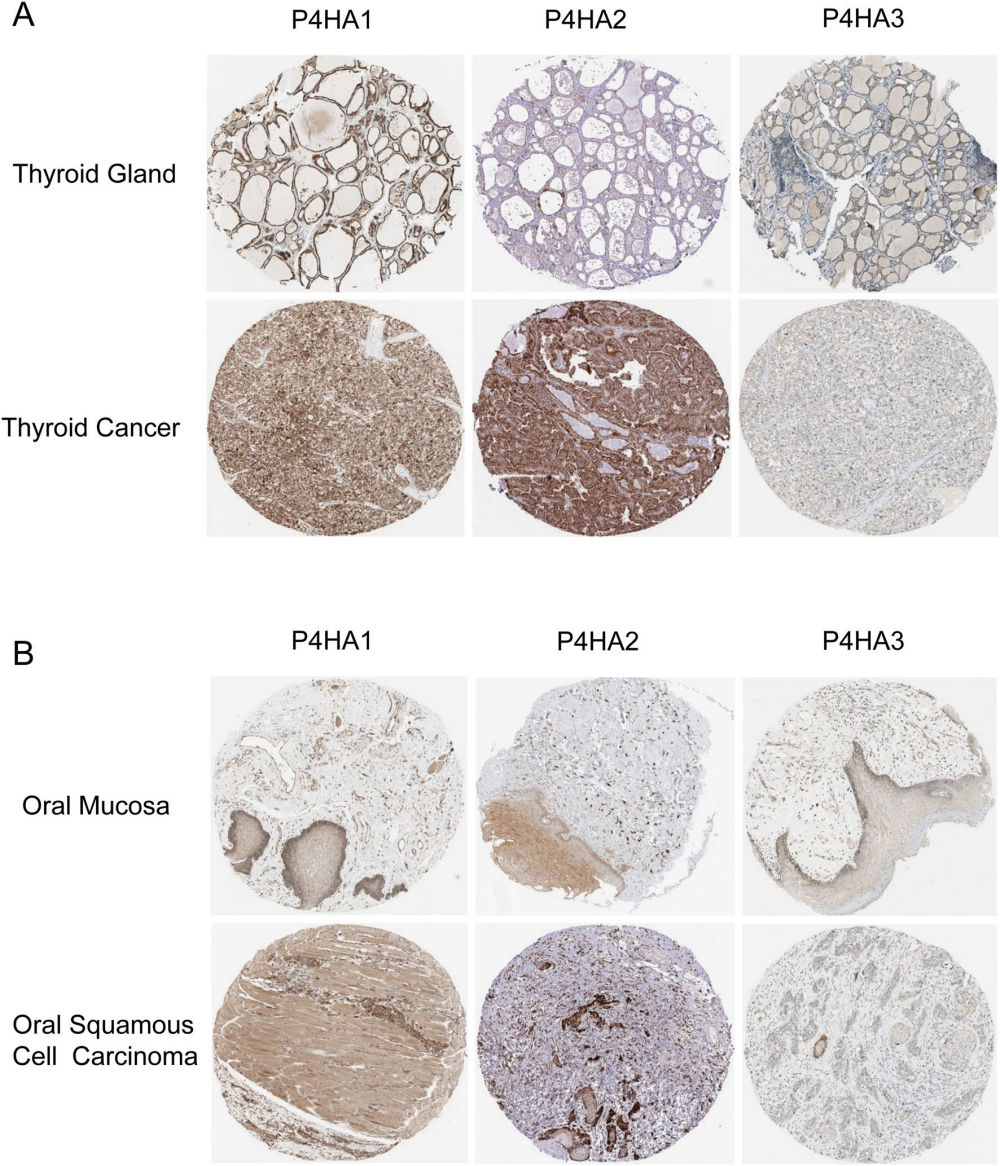
The protein expressions of P4Hs in thyroid cancer and oral squamous cell carcinoma were analyzed by The Human Protein Atlas. (**A**) Expression of P4Hs in normal thyroid tissue and thyroid cancer. (**B**) Expression of P4Hs in normal oral mucosa and oral squamous cell carcinoma.

We further analyzed the protein expression of P4Hs in pathological specimens of HNSC. We found that P4HA1–3 proteins were more highly expressed in thyroid carcinoma tissue than in normal thyroid tissues (Fig. 4A). We also found that the expression of P4HA1–2 proteins was higher in oral squamous cell carcinoma tissues than in normal oral mucosal tissues. The expression of P4HA3 protein was low in both oral squamous cell carcinoma and normal oral mucosa (Fig. 4B).

### Relationship between the P4Hs expression and prognosis in HNSC

**Figure 5:**
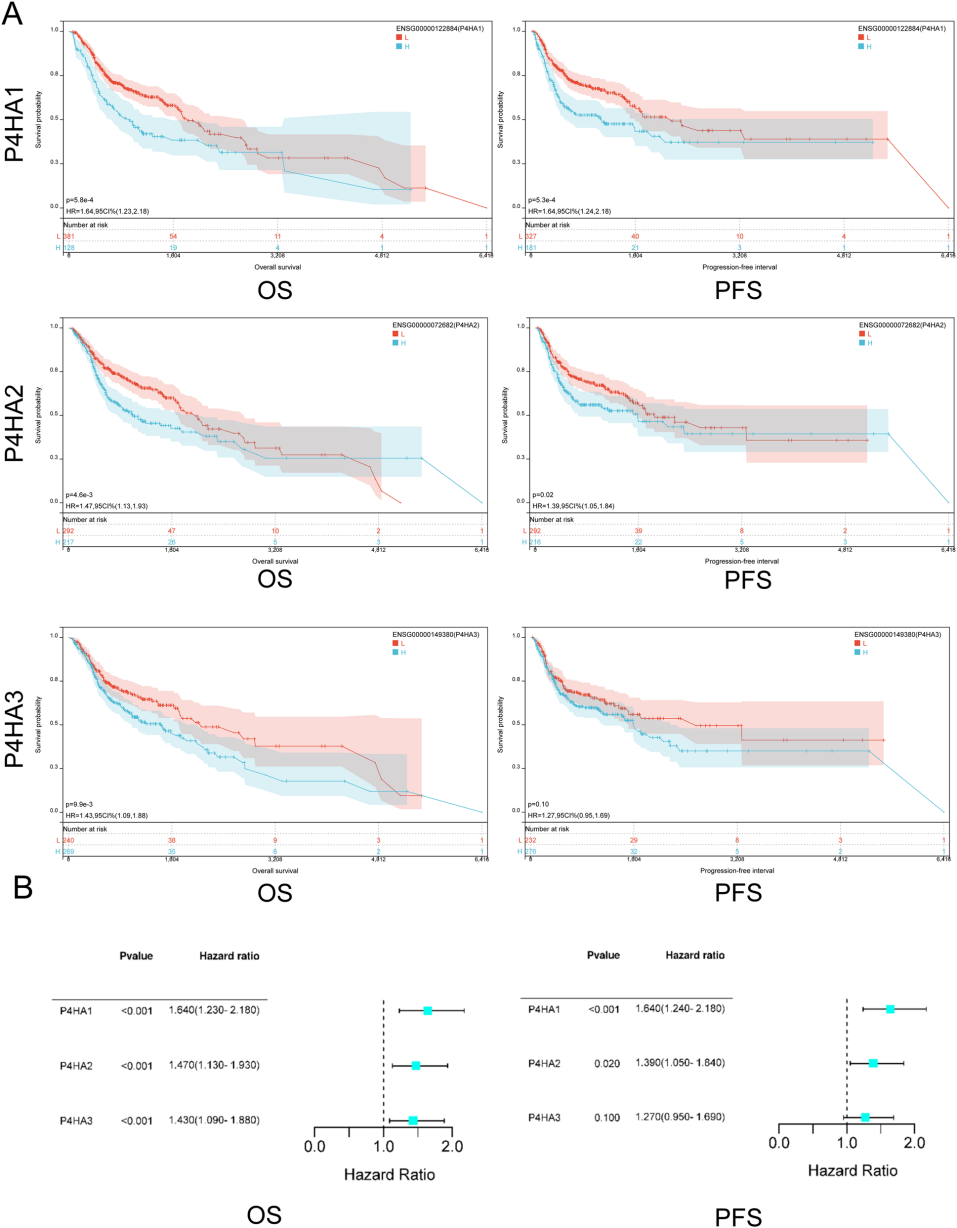
The Prognostic Value of mRNA Level of P4Hs in HNSC Patients. (**A**) Kaplan–Meier curves are plotted to predict the OS and PFS of HNSC patients. (**B**) The Kaplan–Meier curves plotted the risk tree of P4Hs expression for poor prognosis in HNSC patients.

We used Kaplan–Meier plotting tools to further investigate the relationship between P4H expression level and HNSC prognosis. Interestingly, increased P4HA1, P4HA2, and P4HA3 mRNA levels were significantly associated with OS and PFS (*p* < 0.05) (Fig. 5A, B) in patients with HNSC. Patients with high mRNA levels of P4HA1, P4HA2, and P4HA3 were predicted to have low OS and PFS. Conversely, low mRNA levels of P4HA1, P4HA2, and P4HA3 correlated with high OS and PFS.

### Analysis of genetic alteration and function of P4Hs in HNSC

**Figure 6:**
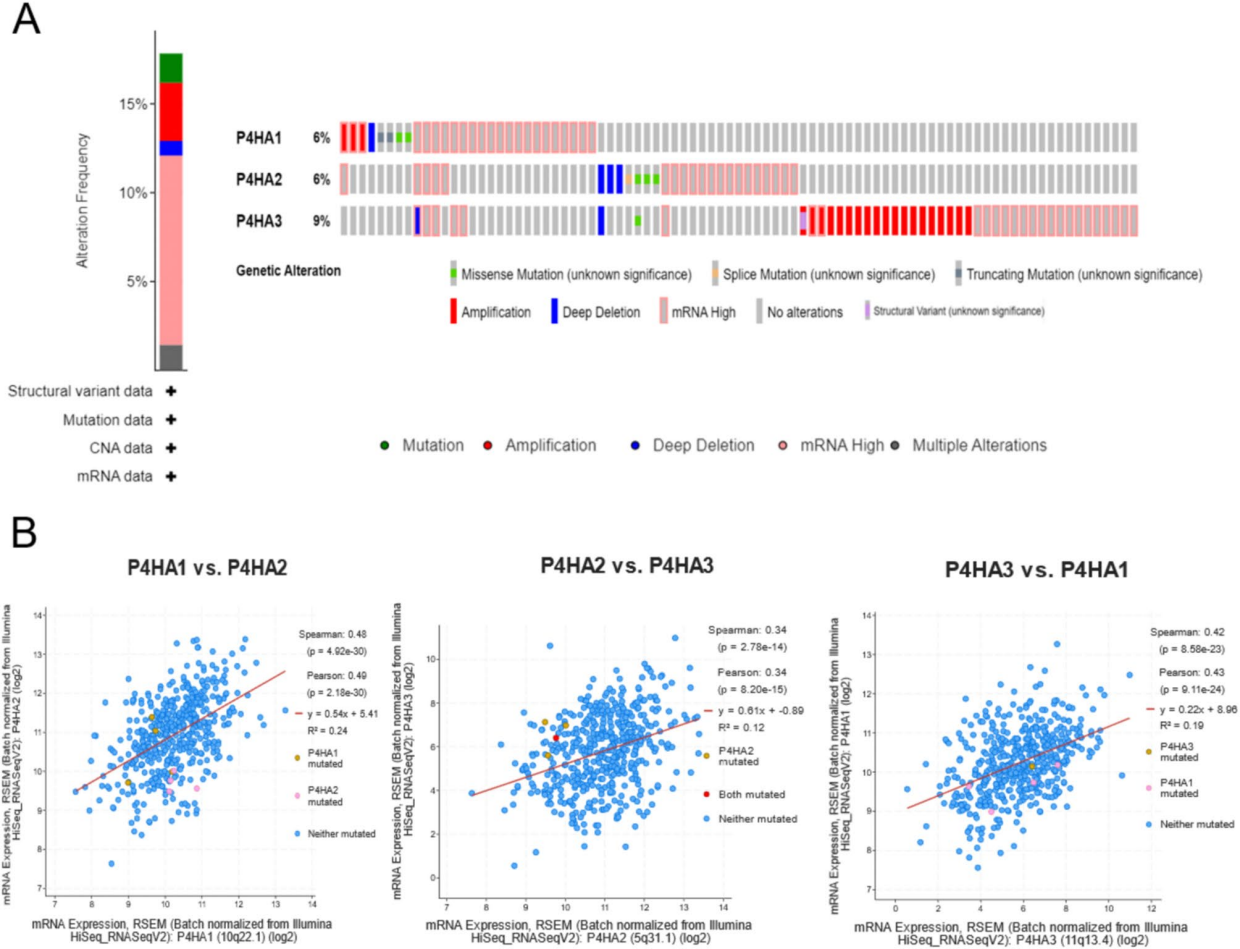
P4Hs Gene Expression and Mutation Analysis in HNSC. (**A**)The expression and mutation of P4Hs gene in HNSC were analyzed by cBioportal. (**B**) Correlation between P4Hs genes was analyzed using cBioportal.

We analyzed P4Hs alterations and correlations using the cBioPortal online tool for HNSC. P4Hs were altered in 28 samples of 488 patients with HNSC (5.74%) (Fig. 6A). We also analyzed the correlations of P4Hs with each other. The results showed that P4HA1 was significantly and positively associated with P4HA2 and P4HA3. In addition, P4HA2 was positively correlated with P4HA1 and P4HA3, and P4HA3 was positively correlated with P4HA1 and P4HA2 (Fig. 6B).

**Figure 7:**
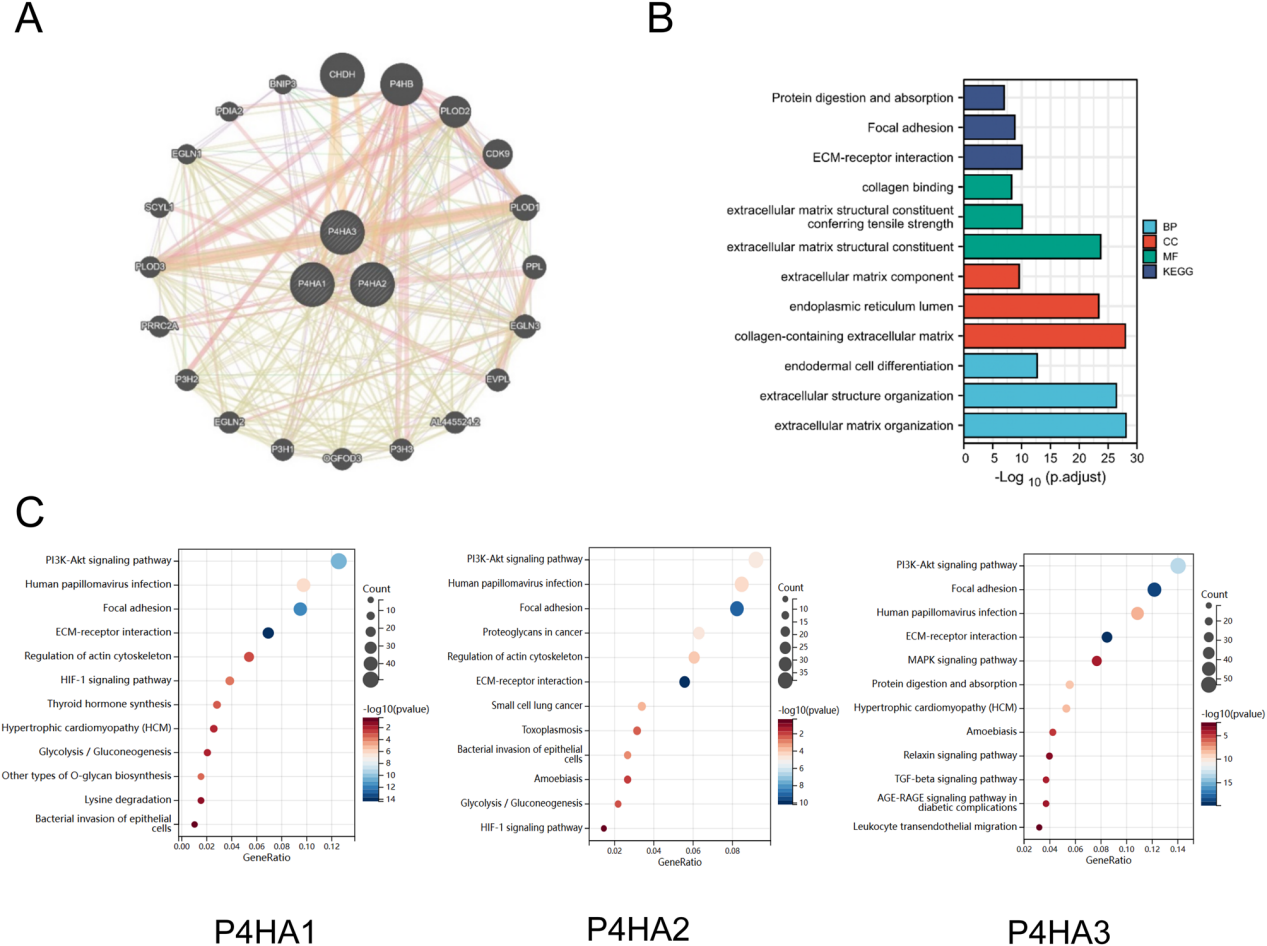
P4Hs significantly related genes and P4Hs function. (**A**) P4Hs and the network of 20 most similar genes were analyzed by Genemania. (**B**) GO and KEGG enrichment of P4Hs and similar genes were analyzed using the Xiantao Academy tool. (**C**) KEGG functional enrichment analysis of P4Hs and associated genes was performed by the SangerBox tool.

We also analyzed P4H networks via Sangerbox and built a network for P4Hs and the 20 most similar genes. The results showed that CHDH, P4HB, PLOD2, CDK9, and PLOD1 were closely related to P4H alterations (Fig. 7A). Furthermore, we predicted the functions of P4Hs by analyzing gene ontology (GO) and KEGG^29–31^ and found that the extracellular matrix structural constituent, endoplasmic reticulum lumen, collagen-containing extracellular matrix, extracellular structure organization, and extracellular matrix organization were significantly regulated by P4H alterations in HNSC (Fig. 7B). Further analysis with Sangerbox shows that P4HA1 is involved in focal adhesion, the PI3K-Akt signaling pathway, actin cytoskeleton regulation, lysine degradation, the HIF-l signaling pathway, glycolysis/gluconeogenesis, and other types of O-glycan biosynthesis. P4HA2 is significantly associated with proteoglycans in cancer, human papillomavirus infection, PI3K-Akt signaling pathway, HIF-l signaling pathway, and glycolysis/gluconeogenesis. P4HA3 regulates focal adhesion, human papillomavirus infection, and the PI3K-Akt, TGF-beta, and MAPK signaling pathways (Fig. 7C).

### P4Hs were correlated with immune cell infiltration in HNSC

**Figure 8:**
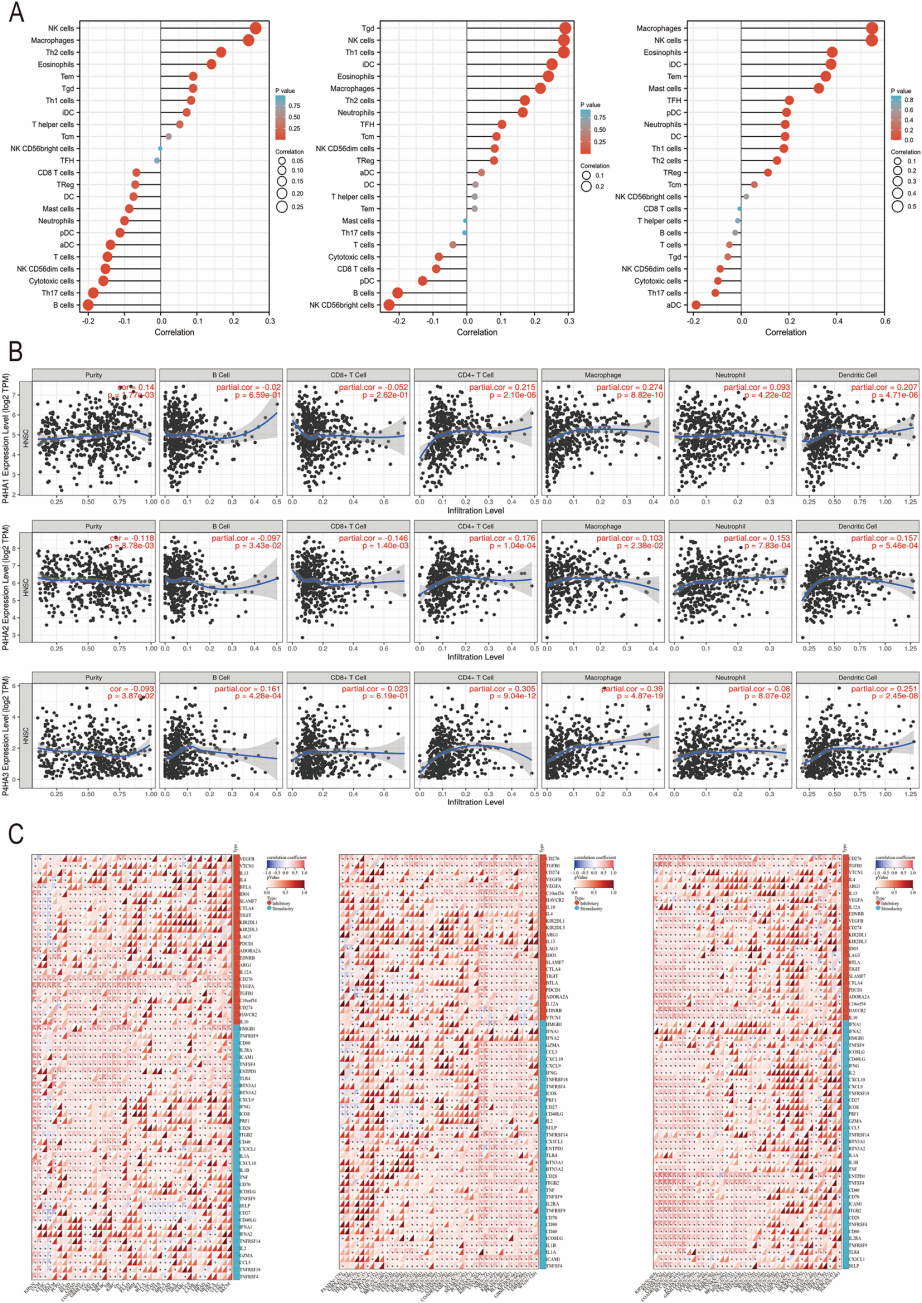
The expression of P4Hs in HNSCS is correlated with the level of immune infiltration and the expression of immune checkpoints (**A** The Xiantao Academy tool; **B** Tumor Immune Estimation Resource; **C** The Sangerbox tool).

Notably, P4H expression was significantly associated with the degree of multiple immune cell infiltration in HNSC (Fig. 8A). We evaluated the correlation between P4H expression and specific cell subsets, including B cells, CD8 + T cells, CD4 + T cells, macrophages, neutrophils, and DC cells (Table S4-6). The analysis results showed that P4HA1 was positively correlated with neutrophils, CD4 + T cells, macrophages, and DC cells in HNSC. P4HA2 was negatively correlated with B cells and CD8 + T cells but positively correlated with CD4 + T cells, macrophages, neutrophils, and DC cells. P4HA3 was positively correlated with B cells, CD4 + T cells, macrophages, and DC cells (Fig. 8B). Further analysis showed that P4Hs were positively associated with most immune checkpoints in HNSC (Fig. 8C). In conclusion, the expression of P4HAs is related to immune cell infiltration and checkpoint gene expression, which indicates that P4HAs can be used as a novel marker for immunotherapy.

### Expression of P4Hs in clinical specimens and its role in tumor progression

**Figure 9:**
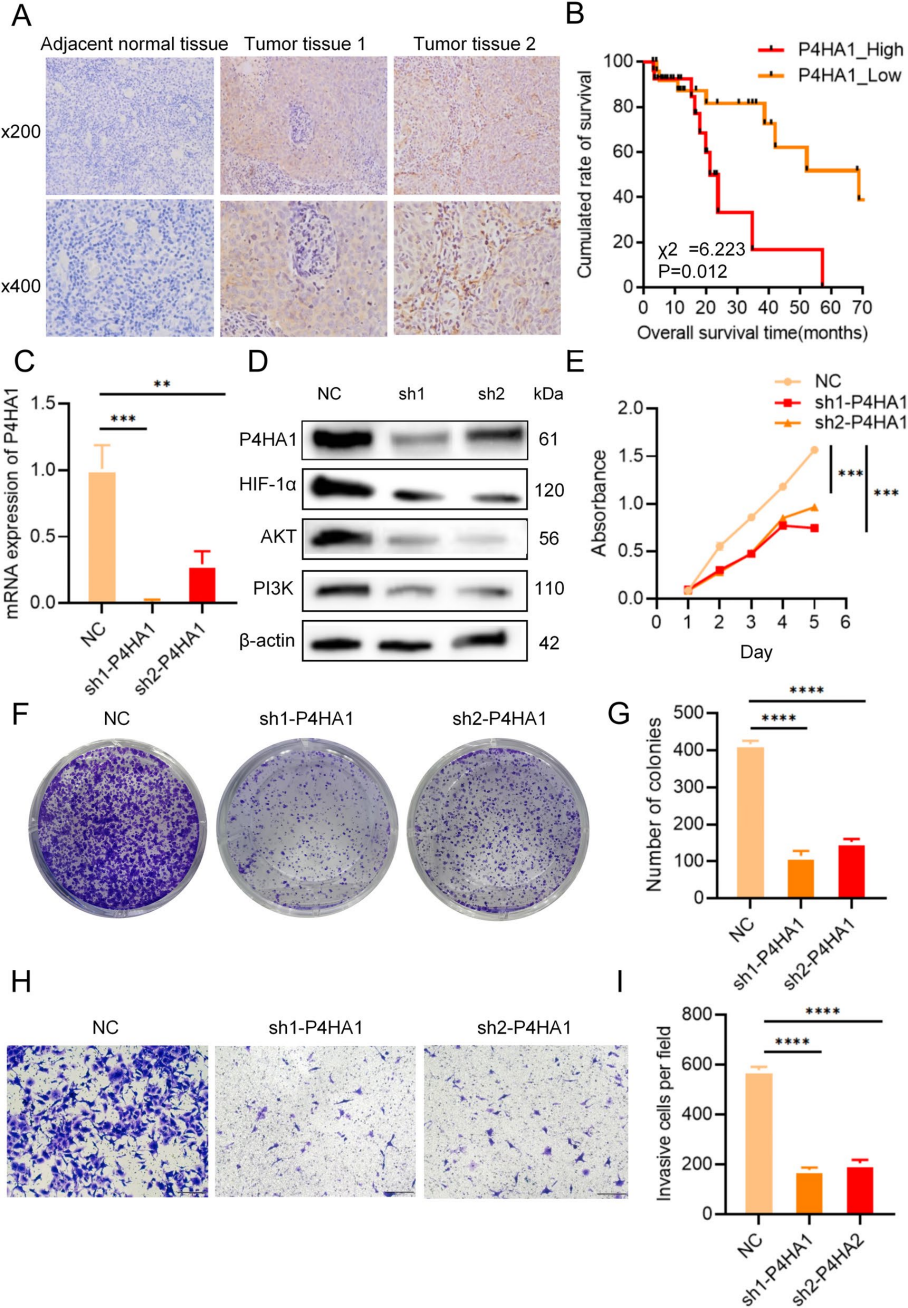
P4HA1 is highly expressed in nasopharyngeal carcinoma tissues and promotes the proliferation and metastasis of nasopharyngeal carcinoma. (**A**) P4HA1 is highly expressed in nasopharyngeal carcinoma. (**B**) Kaplan–Meier curves showed that the high P4HA1 expression group had worse prognosis. (**C**) P4HA1 expression in 5-8F cells were verified by real-time qPCR. (**D**) P4HA1,AKT, HIF-1α, and PI3K expression in 5-8F cells were verified by western blot assay. (**E**) CCK-8 assay showed that P4HA1 could promote the proliferation of nasopharyngeal carcinoma cells. (**F**, **G**) Colony formation assay showed that P4HA1 could promote the proliferation of nasopharyngeal carcinoma cells. (**H**, **I**) Transwell assay showed that P4HA1 could promote nasopharyngeal carcinoma metastasis.

Given that P4HA1 is the major subtype of P4Hs^3,32^, we further investigated the expression of P4HA1 in clinical specimens and its role in tumor progression. First, we used immunohistochemistry to verify the expression of P4HA1 in clinicopathologic specimens of nasopharyngeal carcinoma. Immunohistochemical results were consistent with those from previous bioinformation analysis, and P4HA1 expression was significantly increased in tumor tissues compared with adjacent normal tissues (Fig. 9A). We divided 52 clinical pathological samples of nasopharyngeal carcinoma into P4HA1 high expression group and P4HA1 low expression group. Kaplan–Meier curves showed that P4HA1 high expression group had worse prognosis (Fig. 9B). In addition, to further determine the role of P4HA1 during cancer progression, we performed a correlation experiment using lentivirus-mediated knockdown systems in 5-8F cells. The expression levels of P4HA1 in 5-8F cells were verified by real-time qPCR and western blot assays (Fig. 9C, D). Compared with the control group, the expression levels of AKT, HIF-1α and PI3K were significantly decreased after P4HA1 knockdown (Fig. 9D). CCK-8 and colony formation assays showed that P4HA1 could promote the proliferation of nasopharyngeal carcinoma cells (Fig. 9E, F, G). Transwell assay results showed that P4HA1 played an important role in promoting metastasis of nasopharyngeal carcinoma cells (Fig. 9H, I).

Based on our results, we concluded that P4HA1 is a pro-oncogene that is highly expressed in nasopharyngeal carcinoma and plays an important role in mediating cell proliferation and metastasis.

## Discussion

Previous studies have shown that P4H expression is associated with the progression of multiple cancers and are significantly related to poor prognosis in patients^33–36^. However, at present, the expression and function of P4Hs in HNSC has not been well studied. Further bioinformatics analysis of P4Hs in HNSC has yet to be performed. In this study, we explored the expression of P4Hs in HNSC and its relationship with prognosis and tumor immunity infiltration for the first time. The bioinformatics results suggest that P4HA1-3 was overexpressed in HNSC tumor tissues when compared with corresponding normal tissues. In patients with HNSC, P4HA1 expression was associated with tumor stage, while P4HA2-3 was not. The high expression level of P4HA1-3 was related to worse OS and PFS in HNSC.

Xiaoqi Zhang et al. applied weighted gene co-expression network analysis and found that turquoise and brown modules are closely related to the occurrence of oral squamous cell carcinoma, which may be related to extracellular matrix, intercellular adhesion, and collagen catabolic processes. In addition, Xiaoqi Zhang et al. further identified 10 hub genes such as MMP1, FSCN1, and PLAU as related to oral squamous cell carcinoma^37^.

In the current study, P4HA1 is the most studied isoform of P4Hs. Evidence indicates that P4HA1 is involved in HIF-1α signaling pathway mediated drug resistance in breast cancer^1^. Consistent with reports in the literature, we found that P4HA1 also regulates the HIF-1α signaling pathway in HNSC through bioinformatics analysis. P4HA1-3 was found to co-participate in focal adhesion, the PI3K-Akt signaling pathway, and ECM-receptor interaction in HNSC. P4HA1 was associated with glycolysis in HNSC. Additionally, P4HA3 was related to the TGF-β and MAPK signaling pathways in HNSC. The research results of Xiaoqi Zhang et al. and our analysis provided a reference for further clarifying the pathogenesis of HNSC. Targeting hub genes and related signaling pathways is expected to be a new direction for HNSC therapy in the future. Despite our findings, further investigation is required to fully elucidate the function of P4Hs in HNSC.

The relationship between P4Hs and tumor immunity has not been well studied. Our analysis revealed that P4HA1 was positively correlated with macrophages, while CD8 + T cells were negatively correlated. In addition, P4HA2 was negatively correlated with CD8 + T cells. Previous studies have shown that CD8 + T cells can mediate anti-tumor immunity and inhibit tumor progression^38,39^. These results suggest that P4HA expression is related to immune cell infiltration and checkpoint gene expression and may promote HNSC progression, which indicates that P4Hs may be a possible target of tumor immunotherapy in future research.

We also conducted additional experiments to verify our results. As nasopharyngeal carcinoma is one of the most common head and neck tumors^40^, we detected the expression of P4Hs in pathological tissue samples from patients with nasopharyngeal carcinoma and selected nasopharyngeal carcinoma cell lines 5-8F cells to verify the function of P4Hs. Immunohistochemistry analysis revealed that P4HA1 was highly expressed in nasopharyngeal carcinoma tissues, which is consistent with bioinformatics analysis results. After P4HA1 knockdown, the expression levels of AKT, HIF-1α and PI3K were significantly decreased. Correlation experiments also confirmed that P4HA1 can promote the proliferation and metastasis of nasopharyngeal carcinoma.

In conclusion, P4Hs may be a potential prognostic and immunotherapeutic marker in HNSC, thus supporting new directions for HNSC research and treatment. However, there are some limitations to this study. First, although we conducted relevant experiments to verify our analysis, we only verified the expression and function of P4HA1, not P4HA2–3. In addition, the relationship between P4Hs and immune cell infiltration has not been experimentally verified. Second, although our findings suggest that P4Hs expression is associated with the prognosis and various immune cell infiltration in HNSC, we still cannot confirm that P4Hs affect patient survival through immune infiltration. We only performed survival analyses in HNSC patients, and we do not yet have a clear relationship between p4H2 expression and the efficacy of immune checkpoint inhibitors, which will be the focus of our future research efforts.

Nevertheless, we believe that future studies will further elucidate the function and clinical value of P4Hs in HNSC. Targeting P4Hs in HNSC may be a useful novel anti-tumor strategy in the near future.

### Supplementary Information

Supplementary Information.

## Data Availability

The datasets used or analyzed during the current study are available from the corresponding author upon reasonable request.
