# Supplementary material for: Comprehensive analysis revealed P4Hs as new biomarkers for prognosis and immunotherapy in head and neck cancer
Source: Sci Rep. 2024 May 28;14:12234. doi: 10.1038/s41598-024-62678-9 (PMC11133445; doi:10.1038/s41598-024-62678-9)
Supplement: Supplementary file 1 — Supplementary Information. [file 41598_2024_62678_MOESM1_ESM.docx]

**Supplement materials**

**Table S1. Sequence information used in this study**

| Name | Sequence |
| --- | --- |
| P4HA1 NC | 5’- UUCUCCGAACGUGUCACGUTT -3’ |
| P4HA1 shRNA1 | 5’-AUAUACCAGAUCAUCUUGGAA -3’ |
| P4HA1 shRNA2 | 5’-UAAUAUAUACCAGAUCAUCUU -3’ |

**Table S2. The qPCR primers used in this study**

| Gene |  | Primer Sequence |
| --- | --- | --- |
| P4HA1 | Forward | 5’-AAAAGTGCCTGGCTCTCTGG-3’ |
|  | Reverse | 5’-TGGCTCATCTTTCCGTGCAA -3’ |
| GAPDH | Forward | 5’-GGAGTCCACTGGCGTCTTCA -3’ |
|  | Reverse | 5’-GTCATGAGTCCTTCCACGATACC -3’ |

**Table S3. Information on antibodies used in this study**

| Antibody | Cat. No. | WB | IHC | Specificity | Source |
| --- | --- | --- | --- | --- | --- |
| P4HA1 | 12658-1-AP | 1：4000 | 1：100 | Rabbit | Proteintech Antibody |
| AKT | 10176-2-AP | 1：2000 |  | Rabbit | Proteintech Antibody |
| HIF1α | 20960-1-AP | 1：2000 |  | Rabbit | Proteintech Antibody |
| β-actin | 20536-1-AP | 1：2000 |  | Rabbit | Proteintech Antibody |
| GAPDH | 10494-1-AP | 1：5000 |  | Rabbit | Proteintech Antibody |

**Table S4. The correlation between P4HA1 and marker genes of immune pathways in HNSC.**

| Variable | Partial.cor | p |
| --- | --- | --- |
| Purity | 0.140432502 | 0.001773853 |
| B Cell | -0.020276712 | 0.659019091 |
| CD8+ T Cell | -0.051605231 | 0.262157562 |
| CD4+ T Cell | 0.214549267 | 2.10E-06 |
| Macrophage | 0.27424581 | 8.82E-10 |
| Neutrophil | 0.092871322 | 0.042187058 |
| Dendritic Cell | 0.206774841 | 4.71E-06 |

**Table S5. The correlation between P4HA2 and marker genes of immune pathways in HNSC.**

| Variable | Partial.cor | p |
| --- | --- | --- |
| Purity | -0.117905222 | 0.008782126 |
| B Cell | -0.097055467 | 0.034265551 |
| CD8+ T Cell | -0.146315089 | 0.001401698 |
| CD4+ T Cell | 0.176234957 | 0.000103827 |
| Macrophage | 0.102817328 | 0.023834899 |
| Neutrophil | 0.152955298 | 0.000783307 |
| Dendritic Cell | 0.156889265 | 0.000546402 |

**Table S6. The correlation between P4HA3 and marker genes of immune pathways in HNSC.**

| Variable | Partial.cor | p |
| --- | --- | --- |
| Purity | -0.093124675 | 0.038739058 |
| B Cell | 0.160796592 | 0.000428487 |
| CD8+ T Cell | 0.022883843 | 0.619212923 |
| CD4+ T Cell | 0.304693987 | 9.04E-12 |
| Macrophage | 0.390449148 | 4.87E-19 |
| Neutrophil | 0.079875471 | 0.080744289 |
| Dendritic Cell | 0.25060644 | 2.45E-08 |

Original images:


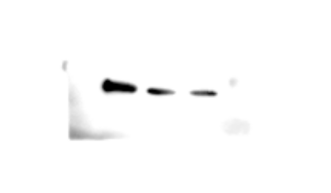
Western blot assays :


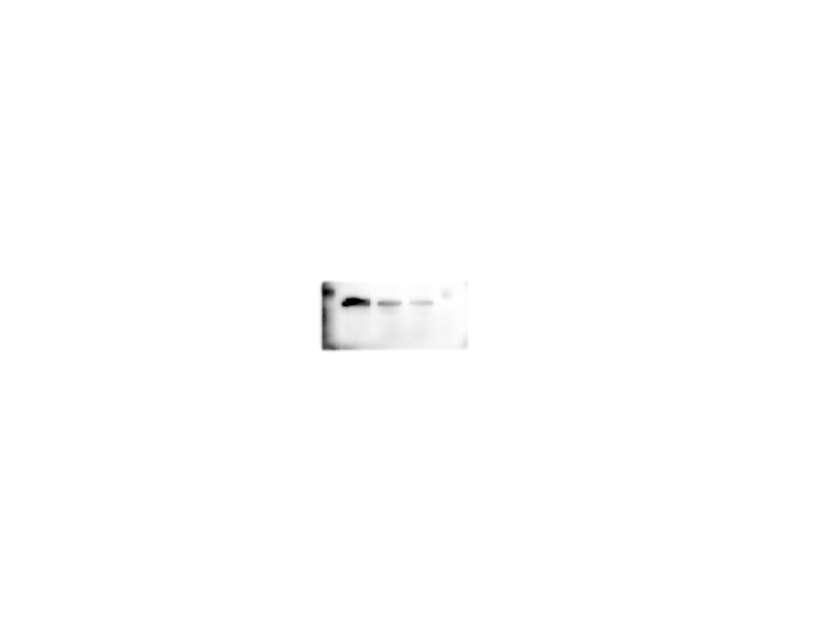

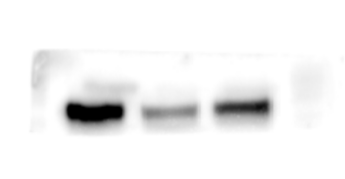


P4HA1 AKT HIF-1α


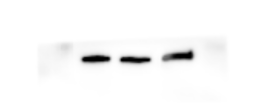

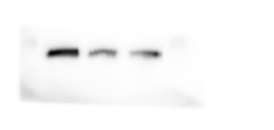


PI3K β-actin

Immunohistochemistry:


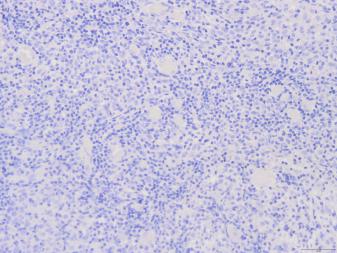

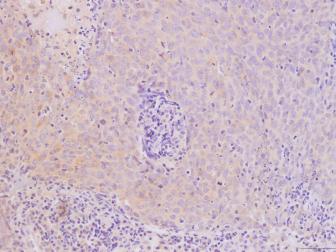

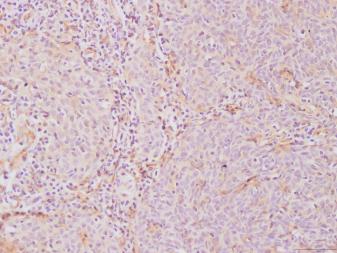


Adjacent normal tissue (x200) Tumor tissue 1 (x200) Tumor tissue 2 (x200)


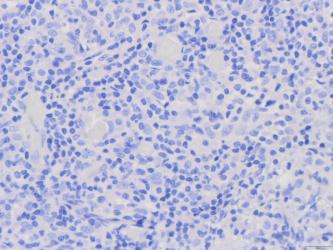

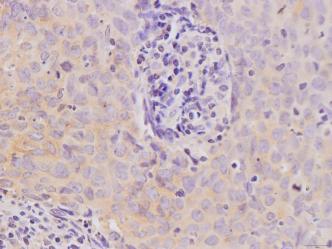

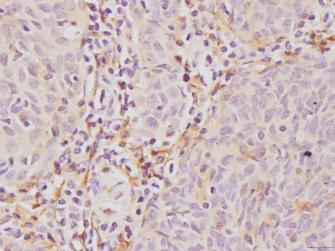


Adjacent normal tissue (x400) Tumor tissue 1 (x400) Tumor tissue 2 (x400)

Colony formation assays:


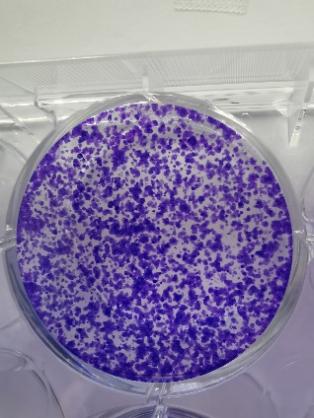

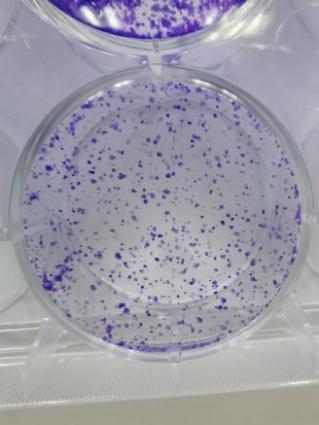

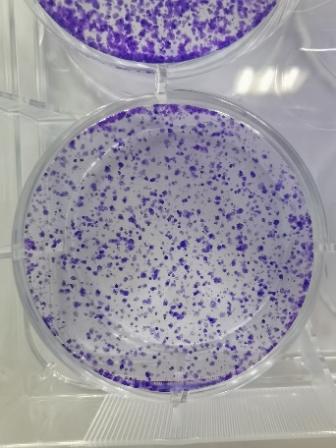


NC sh1-P4HA1 sh2-P4HA1

Transwell assay：


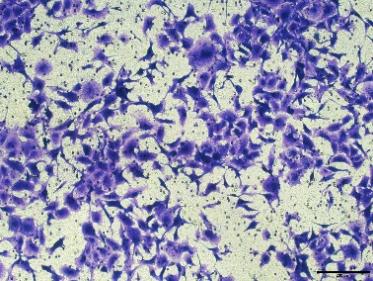

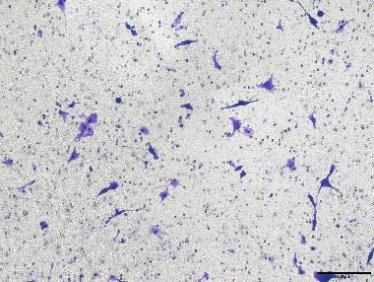

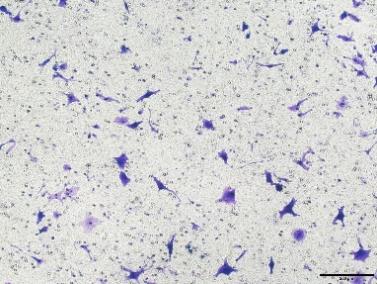


NC sh1-P4HA1 sh2-P4HA1
